# In vivo micro-computed tomography imaging in liver tumor study of mice using Fenestra VC and Fenestra HDVC

**DOI:** 10.1038/s41598-022-26886-5

**Published:** 2022-12-27

**Authors:** Ming Jia Tan, Nazarine Fernandes, Karla Chinnery Williams, Nancy Lee Ford

**Affiliations:** 1 Department of Medicine, The University of British Columbia, Vancouver, Canada; 2 Faculty of Pharmaceutical Sciences, The University of British Columbia, Vancouver, Canada; 3 Department of Oral Biological and Medical Sciences, The University of British Columbia, Vancouver, Canada; 4 Department of Physics and Astronomy, The University of British Columbia, Vancouver, Canada

**Keywords:** Cancer imaging, Experimental models of disease, Computed tomography

## Abstract

Contrast agents are used to enhance the visibility of rodent organs during in vivo micro-computed tomography imaging. Specifically, this non-invasive technique can study liver tumor growth and progression in small animals. Fenestra VC and the novel Fenestra HDVC were compared for enhancement in the liver of healthy and tumor-bearing mice, and the images were compared for their ability to define the tumor border, volume and quantity of tumors. Fenestra VC and Fenestra HDVC were injected into healthy eight-week-old female mice (C57BL/6) via the tail vein then imaged at seven different time points. The experimental results showed that 0.005 mL/g of Fenestra HDVC resulted in the same enhancement for all eight organs as 0.01 mL/g of Fenestra VC across all time points. For the tumor study, B16F10 tumors were surgically introduced into ten eight-week-old female mice (C57BL/6) then imaged in vivo over a 3 day period. Ex vivo micro-CT images of the excised livers were also obtained. The tumor volume and quantity were measured in each image, and the tumour progression observed over 3 days. We showed Fenestra HDVC is effective for in vivo imaging in rodents because the optimal enhancement level in organs is maintained at a reduced injection volume.

## Introduction

The usage of micro-computed tomography (micro-CT) for small animal imaging has been designed to obtain high-resolution images of the animal’s anatomical features^1^. Through the use of in vivo imaging, X-ray beams pass through the body at various angles so that two-dimensional projections can be reconstructed into three-dimensional volumes for visualization^2–4^. The entire imaging process can be completed in a matter of minutes in a noninvasive manner making it possible to conduct long term studies of disease progression in an animal model. One of the limitations for micro-CT imaging is its lack of soft tissue differentiation. This makes it difficult to identify or analyze individual soft tissues and organs in detail. To overcome this limitation, contrast agents (CAs) are used for enhancement.

In general, contrast agents can be categorized into two branches, blood pool or targeting. The primary difference between the two is the organ that is being enhanced. Blood pool CAs will move through the vasculature structures and enhance the blood vessels in each organ as the contrast filled blood passes through before its elimination from the body. In comparison, targeting CAs are labeled with attachments that bind to specific extracellular matrix proteins or cellular receptors in the cells of the organ of interest^3,5^. This will allow the CA to travel through the vasculature then accumulate in the organ of interest and increase its enhancement.

One of the early liver targeting CA, Fenestra LC made in 1995, is a hepatocyte-selective iodinated CA encased in a lipid core to allow the uptake by apoE receptors in healthy hepatocytes^6,7^. This CA was made from the lipid component 2-oleoylglycerol 1,3-bis[7-(3-amino-2,4,6-triiodophenyl) heptanoate] with a mean particle diameter less than 150 nm and have an iodine concentration of 50 mg/mL^7^. It is known that Fenestra LC has a short circulation time of roughly 4 days and has a high injection volume between 200 and 400 µL for a 20 g mouse^6,8^. This limits the possibility to conduct long term liver studies in mice because the small animals would require repeated injections^6,8^. In the early 2000s Fenestra VC a lipid-based iodinated, hepatoselective blood pool CA was produced^9^. It was made from the same lipid component, particle size and iodine concentration as Fenestra LC so when Ford et al.^10^ conducted a time course study using Fenestra VC they discovered that it had dual-imaging properties. Fenestra VC behaved like a blood pool CA for the first few hours immediately after injection into the mouse, then as it was being eliminated through the hepatobiliary pathway it behaved like a liver targeting CA^10^. In addition, the blood pool CA characteristic of Fenestra VC also contributed to its prolonged circulation time as compared to Fenestra LC^6^.

Even though Fenestra VC has dual-imaging properties and an iodine concentration of 50 mg/mL, the amount injected into a 20 g mouse is relatively high ranging between 300 and 400 µL^8^. Willekens et al*.*^11^ noted the importance of lowering the injectable volume of CA into small rodents because the total body size of the animal is small so any volume above 0.2 mL for a 20 g mouse may lead to serious health effects including death. The potential side-effect with reducing the injectable CA volume is that the contrast enhancement in the vasculature may also be reduced. So, to overcome the limitations of reducing injectable CA volumes while keeping the enhancement at a desirable level, Fenestra HDVC a novel CA was created, as a concentrated version of Fenestra VC, with an iodine concentration of 100 mg/mL.

In this study, the dual-imaging property of Fenestra HDVC and Fenestra VC will be compared in healthy mice, and used in a tumor progression study. The CAs will be used to monitor and observe the tumor progression and growth (B16F10) in the livers of a wild-type strain of mice (C57BL/6) with a high-resolution in vivo micro-CT scanner. The results will be used to confirm the ability of a more concentrated CA at a lower injection volume to produce the optimal enhancement level in the liver for tumor progression study.

## Results

### Time course study

**Figure 1:**
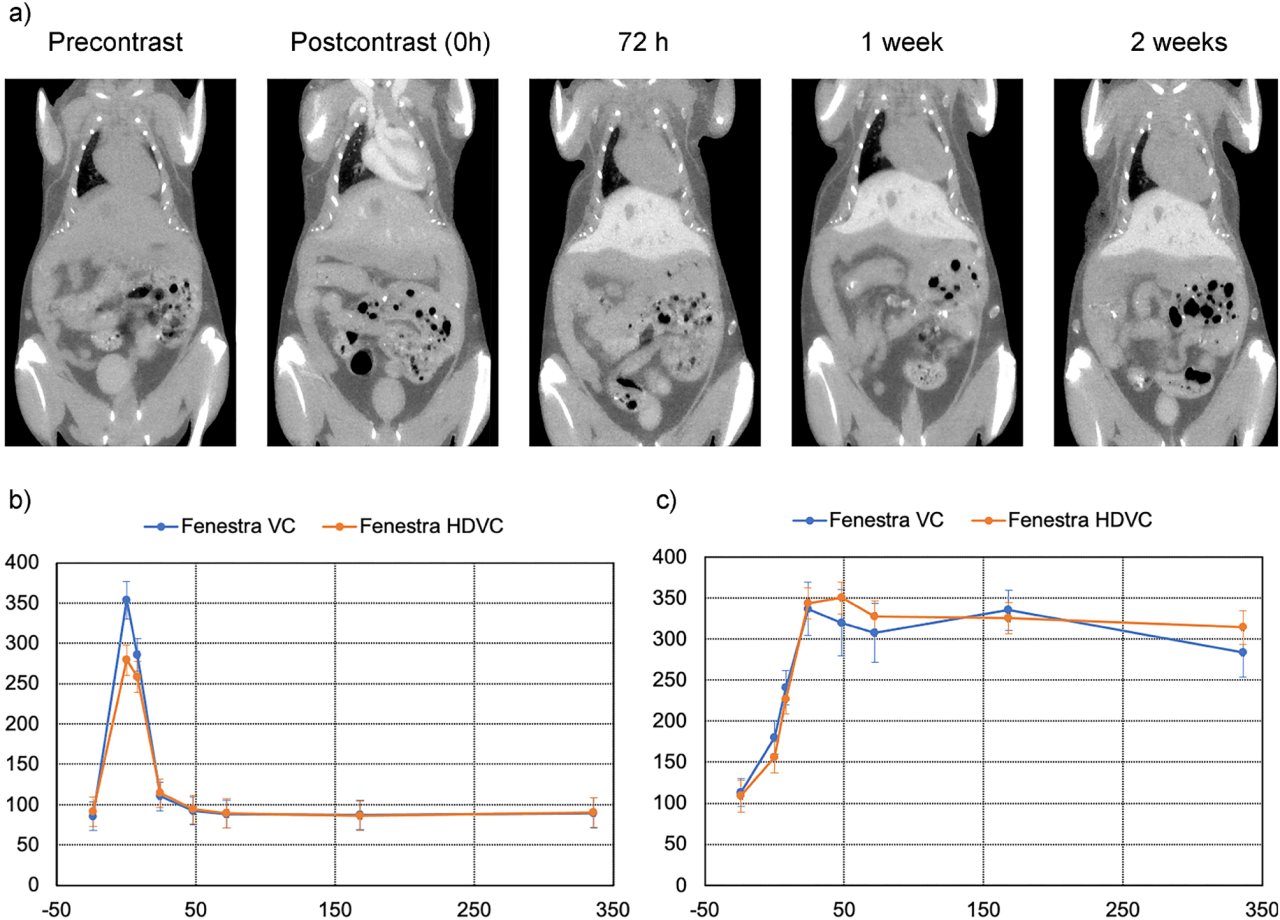
Time-course of contrast enhancement in a single mouse over a 2-week period. (**a**) Images obtained in vivo*,* with an isotropic voxel spacing of 0.1 mm. Average CT number in HU over the same 2-week period measured in the heart (**b**) and the liver (**c**) for Fenestra VC (blue) and Fenestra HDVC (orange). Data points are the mean and standard deviation of 5 mice.

Figure 1 shows the time course of Fenestra HDVC over 2 weeks post-injection. Figure 1a shows the enhancement in the vasculature over the early time points, then uptake in the liver at delayed timepoints. Figure 1b) shows the enhancement curve for the blood and the liver parenchyma, with Fenestra HDVC in orange and Fenestra VC in blue. Although the iodine concentration of the Fenestra HDVC agent (100 mg/mL) was double that of Fenestra VC (50 mg/mL), similar contrast enhancement was achieved by introducing a reduced injected volume of the Fenestra HDVC agent. The average greyscale value and standard deviations of Fenestra HDVC were recorded in Table 1. The right atrium immediately post-injection had a contrast enhancement of 188 HU over the unenhanced greyscale value. The enhancement in the liver at post-contrast 48 h measured 241 HU. The other clearance organs (bladder, kidney, and spleen) did not show large contrast enhancements.

**Table 1:** Average greyscale value and standard deviation for Fenestra HDVC.

Time (h)	Mean Greyscale Value ± Standard Deviation [HU]							
	Right Atrium	Liver	Air	Bladder	Leg Muscle	Kidney	Spleen	Vena Cava
-24	91 ± 19	109 ± 19	−983 ± 16	128 ± 17	127 ± 18	87 ± 15	96 ± 20	–*
0	279 ± 18	156 ± 19	−977 ± 15	154 ± 17	128 ± 18	132 ± 17	165 ± 18	224 ± 28
8	258 ± 19	227 ± 18	−970 ± 17	216 ± 18	154 ± 18	145 ± 17	205 ± 20	230 ± 25
24	114 ± 18	344 ± 19	−970 ± 16	180 ± 21	144 ± 18	178 ± 18	246 ± 20	137 ± 21
48	94 ± 18	350 ± 20	−977 ± 16	162 ± 18	135 ± 18	124 ± 18	276 ± 26	124 ± 25
72	89 ± 18	328 ± 19	−964 ± 17	152 ± 20	132 ± 17	126 ± 17	298 ± 24	121 ± 22
168	86 ± 18	325 ± 19	−956 ± 17	142 ± 19	130 ± 18	124 ± 18	290 ± 28	113 ± 21
336	90 ± 18	314 ± 20	−974 ± 16	167 ± 19	128 ± 18	120 ± 18	277 ± 27	112 ± 21

The mean greyscale values and standard deviations in Hounsfield units for the five mice injected with Fenestra HDVC. Asterisk (*) indicates an inability to locate the vena cava at that specific time point.

### Tumor study

#### In vivo micro-computed tomography images

**Figure 2:**
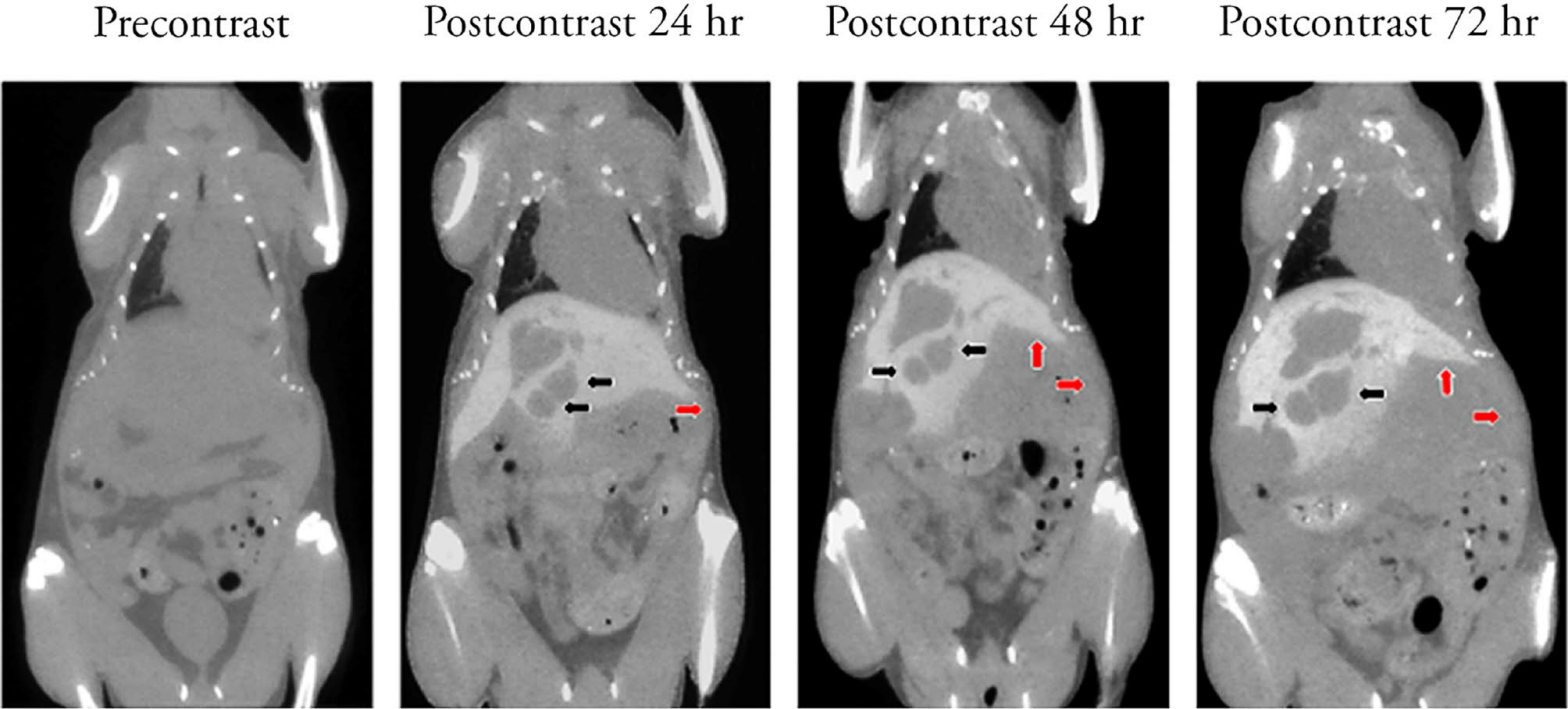
B16F10 tumor growth in the mouse liver observed using Fenestra HDVC. The murine melanoma cells grew in the mouse for two weeks before the mouse was given a single dose of Fenestra HDVC. Using in vivo micro-CT, the quantity and volume of tumors in the liver were measured for three days. The tumors measured for volume are indicated by the black arrows and the abnormal protrusion in the abdominal cavity and the liver is shown by the red arrows.

The in vivo micro-CT image of a mouse given a single dose of Fenestra HDVC two weeks after it received its hepatic portal vein B16F10 murine melanoma cell injection surgery is shown in Fig. 2. The tumors cannot be observed in the pre-contrast image, but they are obvious once the Fenestra HDVC enhances the liver to appear light grey (Fig. 2 black arrows). The CA maintains its contrast enhancement in the liver consistently for 3 days so that the tumor growth can be observed. In this figure the growth of multiple tumors on the left side of the liver expands that side of the liver downwards. The mouse also had an abnormal protrusion under the right side of its liver caused by metastatic tumor nodules that grew large and began to push outwards (Fig. 2 red arrows). The result is a decrease in the contrast-enhanced right liver in the mouse and a bloating of the abdominal region caused by the growth of multiple tumor nodules in different organs and tissues that was visible externally.

#### In vivo and ex vivo tumor analysis

**Figure 3:**
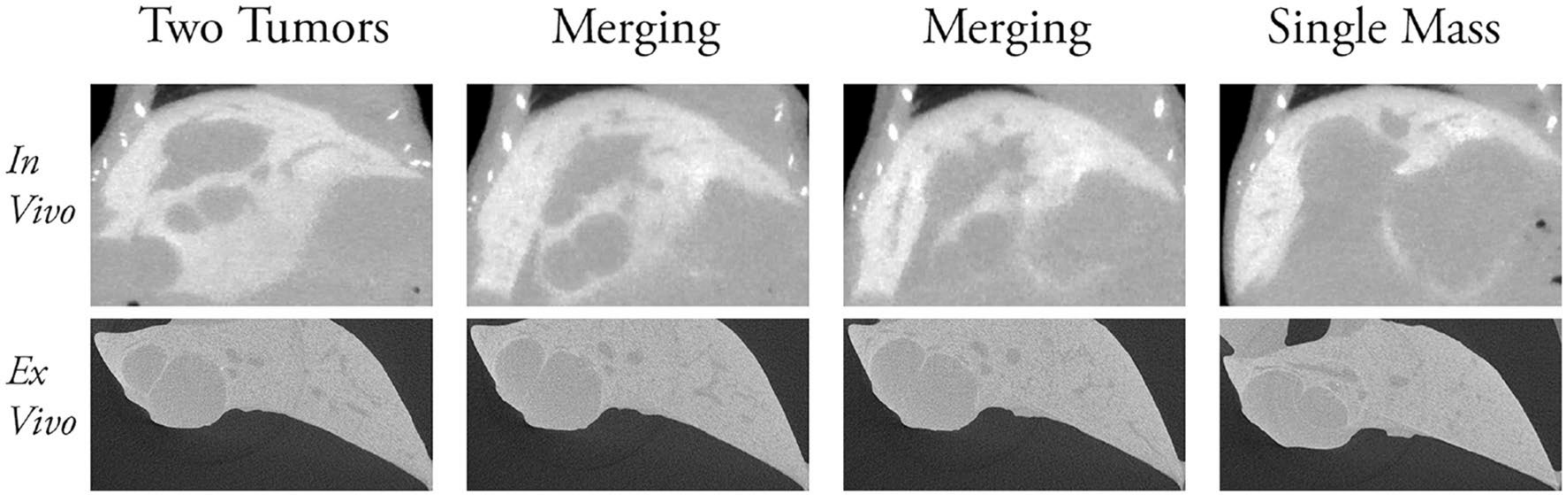
Tumor merging observed on in vivo and ex vivo micro-CT imaging. The merging of two tumors in the liver of the same mouse was compared in the in vivo and the ex vivo micro-CT images. The two separate tumors merged somewhere inside the liver. The merging progression occurs over more slices in the ex vivo image due to higher resolution of this technique.

The excised mouse livers were imaged using the ex vivo micro-CT specimen scanner and compared to the images obtained from the in vivo micro-CT. Both the ex vivo and in vivo images show the tumors as a dark grey mass and the enhanced liver as light grey. The border of the tumors are more defined in the ex vivo images with a sharp line distinguishing tumor from tissue. The same tumor in the in vivo image will have a softer border that is less defined and does not clearly separate the tumor from the tissue. There were situations where two tumors grew close together, but the in vivo images only show one large tumor mass because of the lower resolution. The same tumors in the ex vivo images appeared as separate tumors extremely close together but separated with a thin line of enhanced liver tissue because of its higher resolution. There are also tumors that eventually grow into each other and merge to form one. This merging process can be observed in both images. The two merging tumors in the ex vivo image was shown in detail over a depth of 10 mm in the liver with an initial thin separation that was slowly closed by the growth of the two tumors until they merged together (Fig. 3 bottom row). The in vivo image progresses through the merging process of the same tumors faster; in fewer slices representing the same liver thickness, the two separate tumors fused into one mass (Fig. 3 top row). The in vivo image was able to show the merging process inside the liver but lacked detail that was only observable in the ex vivo image.

#### Tumor volume measurement

**Figure 4:**
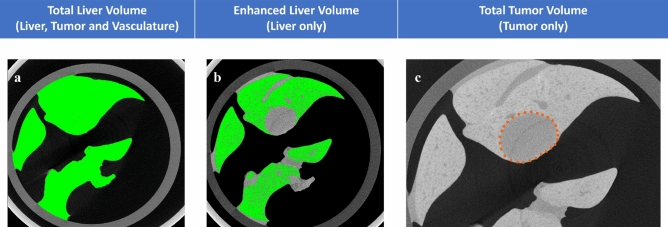
Volume measurements of liver, liver vasculature and tumor from mouse 2. The Total liver volume measured the entire volume of the liver including the liver vasculature and tumor (**a**). The enhanced liver parenchyma only has the volume of the liver without the liver vasculature or tumors (**b**). The manually obtained total tumor volume only has the volume of the tumor from the liver of mouse 2 (**c**).

The total liver volume includes the entire enhanced liver along with the tumors and the vasculature. The enhanced liver parenchyma could be easily segmented. However, the unenhanced region included the tumours, but also the vasculature. The vasculature was estimated from one mouse, and the volume was applied to the entire group, as they were age- and sex-matched. One mouse grew a single well-formed tumor that was completely enclosed within the liver, and was therefore chosen to estimate the liver vasculature. The measured volumes are shown in Fig. 4. The entire liver volume measured 1444.98 mm^3^ (Fig. 4a) and the enhanced liver parenchyma measured 902.75 mm^3^ (Fig. 4b). The difference was 542.23 mm^3^ but this value also included the liver vasculature, so the tumor nodule in the liver was manually measured and the total tumor volume was 51.46 mm^3^ (Fig. 4c). The total vasculature volume (Eq. 1) was calculated to be 490.77 mm^3^ and the percentage of the total tumor burden (Eq. 2) was calculated to be 3.56%. For all the other mice, the adjustment was made by subtracting the vasculature volume of mouse 2 (490.77 mm^3^) from their measured enhanced liver volume shown in (Eq. 2), and the percentage of the total tumor burden for each mouse was calculated. The measured tumour volumes for selected tumours from the in vivo scans, along with the ex vivo volumes and total tumor burden are given in Table 2 for mice that received the Fenestra VC agent, and in Table 3 for the mice that received Fenestra HDVC. In Table 2, mouse 5 did not have any recorded tumor volume in the in vivo images because all the small tumor nodules merged to form one large tumor mass. It was difficult to identify the edge of any individual tumors because the tumor grew on the surface of the liver, but tumors were non-enhanced, so the in vivo image showed a dark grey tumor mass with certain regions surrounded by enhanced liver tissues.

**Table 2:** Tumor quantity and volume measured from in vivo and ex vivo micro-CT images for the five mice injected with Fenestra VC.

	Mouse 2		Mouse 5		Mouse 6		Mouse 7		Mouse 12	
	In Vivo	Ex Vivo	In Vivo	Ex Vivo	In Vivo	Ex Vivo	In Vivo	Ex Vivo	In Vivo	Ex Vivo
Tumor quantity	1	2	8	7–8	4	4	4	2	3	8–9
Tumor volume (mm^3^) Day 1	20.35	–	–	–	19.74	–	14.64	–	21.45	–
Tumor volume (mm^3^) Day 2	35.51	–	–	–	33.75	–	17.53	–	23.56	–
Tumor volume (mm^3^) Day 3	51.07	51.46	–	–	31.34	32.34	19.74	19.26	28.27	32.85
Total tumor burden (%)	–	3.56	–	61.94	–	39.39	–	12.53	–	66.28

The quantity was measured by counting individual tumors in the liver. The tumor volume is measured in the in vivo and ex vivo images.

**Table 3:** Tumor quantity and volume measured from in vivo and ex vivo micro-CT images for the five mice injected with Fenestra HDVC.

	Mouse 3		Mouse 4		Mouse 9		Mouse 10		Mouse 11	
	In Vivo	Ex Vivo	In Vivo	Ex Vivo	In Vivo	Ex Vivo	In Vivo	Ex Vivo	In Vivo	Ex Vivo
Tumor quantity	7–8	11–12	6	7	4	7–8	3	5–6	–	–
Tumor volume (mm^3^) Day 1	8.86	–	35.61	–	1.12	–	12.99	–	8.05	–
Tumor volume (mm^3^) Day 2	10.87	–	57.02	–	1.56	–	20.57	–	14.59	–
Tumor volume (mm^3^) Day 3	14.30	11.74	90.78*	69.15	2.25	2.82	23.93	27.64	16.62	–
Total tumor burden (%)	–	34.90	––-	35.74	–	41.46	–	15.32	–	70.35

The quantity was measured by counting individual tumors in the liver. The tumor volume is measured in the in vivo and ex vivo images. The asterisk (*) indicates a large volume change that does not follow the growth pattern of all other tumors measured.

**Figure 5:**
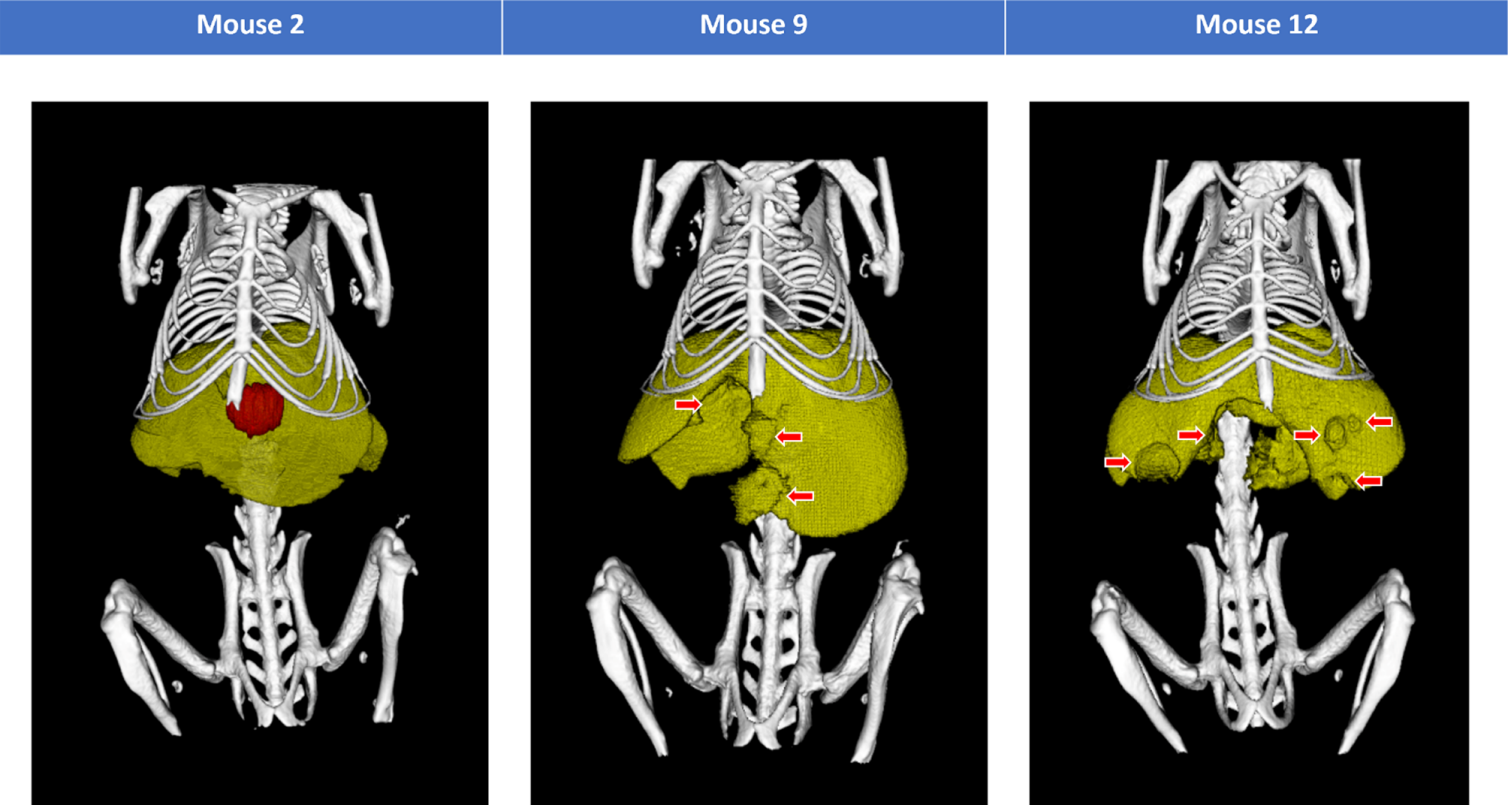
3D models of tumor burden. Mouse 2 with a single tumor nodule (red sphere) fully enclosed in its liver (yellow) is the light tumor burden. Mouse 9 with several tumor nodules (red arrows) that grew inside the liver (yellow) represents the medium tumor burden. Mouse 12 with a tumor mass (red arrow pointing to the large gap in the center of the liver) surrounded by several tumor nodules (red arrows pointing to the spherical indentations) inside its liver (yellow) represents the heavy tumor burden.

The 3D model of mouse 2, mouse 9 and mouse 12 were constructed using the in vivo image from the third day of scanning (Fig. 5). Mouse 2 and mouse 9 were given three weeks to develop tumors before they were imaged while mouse 12 was given two weeks. Only mouse 2 developed a single tumor nodule fully enclosed in the liver so the 3D model of mouse 2 contains the tumor nodule (shown in red). For mouse 9 and mouse 12, they developed multiple tumors and tumor masses that were not fully enclosed inside the liver making it difficult to identify the border of the tumors. The holes in the liver of mouse 9 and mouse 12 indicate the positions at which tumor nodules or tumor masses developed (red arrows in Fig. 5).

## Discussion

Similar to Fenestra VC, the HDVC agent also showed a strong contrast enhancement for the vasculature at early timepoints, with delayed uptake in the liver at 24 h post injection. There were no significant differences (*p* > 0.05) between the two contrast agents for the clearance organs at the eight time points. Similarly, there were no significant differences (*p* > 0.05) for the heart and vasculature. These results confirmed that mice injected with 0.01 mL/g of Fenestra VC or 0.005 mL/g of Fenestra HDVC will have the same amount of iodine introduced into their systems and the contrast enhancement for the eight organs of interest across all the time points were the same.

The time course study has shown that Fenestra HDVC can maintain excellent contrast enhancement. One benefit of Fenestra HDVC is that the higher iodine concentration allows for a reduced injection volume. This will introduce less stress to the cardiac system in the small rodents and lower the chance of potential death^11^. The reduced iodine volume will also bring less stress to the excretory organs. One main difficulty observed was the higher iodine concentration directly increased the viscosity of Fenestra HDVC. This made the CA thicker and more resistant to flow so when drawing up Fenestra HDVC from the vial or injecting it into the tail vein of the mouse the process was more difficult compared to Fenestra VC. The small diameter of the tail vein in the mouse also limits the needle size to 29-gauge so the tail was heated to expand the vein to its maximum diameter in an attempt to reduce some resistance. By having a veterinary technician perform the injections, all mice received the full dose of the contrast agent successfully.

The tumors were not limited to growing in the liver because B16F10 murine melanoma tumors are known to be metastatic. This means that the tumors cells can travel along the circulatory system and settle in other organs and tissues where they grow into new tumor nodules. As demonstrated by other research groups B16F10 cells can metastasize to the lymph nodes, stomach, lungs, brain, bones, and other vital organs^12,13^. The lung is a unique organ that can be monitored for tumor growth using in vivo micro-CT imaging without CAs. As Winkelmann et al.^12^ demonstrated, the tumors that grew in the lungs can be clearly seen in the axial and sagittal position of the mice even when imaged without CAs. Tumors in the bones were also visible without CAs because the bones that were destroyed had swollen local soft tissues^12^. For the liver, it was necessary to use CAs to differentiate it from other soft tissue organs in the abdominal cavity because CAs can enhance the liver’s visibility during the in vivo micro-CT scans.

In Fig. 2, the mouse had brightly enhanced liver parenchyma making the dark tumor nodules and tumor masses stand out. The CA also shows other important features including the expansion of the liver caused by the growing tumors, abnormal protrusions in the abdominal cavity and tumor overgrowth onto the surface of the liver (Fig. 2). This shows that B16F10 cells replicate rapidly leading to a visible change to the shape of the liver and the bloating feature of the abdominal region of the mouse. Additionally, the metastatic characteristic of B16F10 cells caused tumor nodules to grow in other organs and can expand upward to grow over the bottom region of the liver (Fig. 2 red arrow). This resulted in the decrease of both the enhanced visible liver region and the number of functional healthy hepatocytes causing greater physical stress in the animal.

The in vivo micro-CT image was able to show the growth of the tumors with the use of CAs up to post-injection 72 h. In comparison, Boll et al. observed that when using, the alkaline earth metal-based NP CAs, ExiTron nano 6000 and ExiTron nano 12000 for micro-CT scans the peak enhancement time points were at post-injection 30 min and 4 h respectively^6^. ExiTron nano 6000 and ExiTron nano 12000 were shown to enhance the liver from several weeks to months, allowing for repeated micro-CT scans with a single CA injection^6^. Boll et al.^6^ noted that the injection dose for ExiTron nano 12000 was unfavorably high, but it produced higher contrast and had a longer scanning window making it preferable compared to ExiTron nano 6000.

The other CA that Boll et al.^6^ tested was Fenestra LC, the liver targeting CA. Different from the blood pool Fenestra VC or Fenestra HDVC, Fenestra LC directly enhanced the liver upon injection, so it was possible to image the liver at post-injection 30 min whereas we started our first image at post-injection 24 h^6^. The benefit of having a CA with a quick enhancement and long circulation time is that tumors can be detected in its very early stages. As noted by Kim et al.^14^, they immediately detected multiple tumor nodules one week after the tumor injection whereas the previous six days there were no signs of tumor growth.

The in vivo and ex vivo tumor images of the same mouse are compared in Fig. 3, and the results for all mice are shown in Tables 2 and 3. The ex vivo images provide a more detailed view of the tumor to clearly differentiate between a tumor nodule and a liver blood vessel, and the shaper edges around the tumors make it possible to outline a more accurate tumor border. In addition, ex vivo imaging allowed more tumors to be visualized for identification, specifically the small tumors that were close together and seemed like one big tumor in the in vivo images. In the experiments of Grace et al. they discovered that the metastatic B16F10 cells will develop on average 5 tumor nodules in the liver^13^. This was consistent with the number of tumors counted and recorded in Tables 1 and 2 given that the B16F10 cells were introduced into the mice through different methods. These results confirm the metastatic property of B16F10 cells.

In comparison the in vivo images have a less defined edge especially where the tumors protruded out of the liver, so the tumor measurements were less accurate. This was consistent with the report of Kim et al.^14^, where they found that the tumor measurements made on the in vivo micro-CT images tend to be larger than the actual size of the tumor suggesting that in vivo images will overestimate the size of the tumor.

In Table 3, mouse 4 had a different tumor volume on day three for the in vivo and ex vivo images. There are several possible reasons for the volume difference. This was the largest tumor in the liver, so it could have grown out onto the surface of the liver. The thin protective outer layer of the tumor mass was very fragile so during the excision process some sections of the tumor could have been ruptured causing some of the tumor cells to be lost. A second potential reason is the difference in resolution and voxel size for the in vivo and the ex vivo image. The in vivo image was reconsrtucted at 100 µm whereas the ex vivo image was at 17 µm so the measurement error in the in vivo image will result in a larger volume than a measurement error in the ex vivo image. The resolution in the in vivo image is also less than the ex vivo image so the border of the tumor was not as defined. In some cases, there was a gradient of grey surrounding the tumor, forming a fuzzy border between the tumor and the liver. In other situations, the tumor might have grown near the edge of the liver, so it protruded out of the liver but since only the liver was enhanced by the CA, it was difficult to estimate the size of the tumor among the unenhanced tissues. These are some potential reasons that could have resulted in the large tumor volume growth for mouse 4 on day three in Table 2.

## Conclusion

The increased iodine concentration of 100 mg/mL in Fenestra HDVC can lead to many benefits. A smaller injection volume reduces the stress on the heart and excretory organs in the small animal. But since the total amount of iodine introduced into the animal did not change, the optimal enhancement is maintained in the organ of interest. In addition, Fenestra HDVC as a lipid-based iodine CA has also retained the delayed-imaging properties of Fenestra VC. This means that after the injection the vasculature will be enhanced for up to eight hours, then as it passes through the liver for elimination it will accumulate in the liver and enhance it like a liver-targeting CA. This delayed-enhancement was used to monitor the growth of tumours over a 3 day period in vivo following a single injection of the contrast agent. Furthermore, the contrast agent remained in the liver parenchyma following excision of the liver, which enabled higher-resolution scanning ex vivo*.* These advantages suggest that Fenestra HDVC is a safe and effective contrast agent for small animal in vivo and ex vivo micro-CT imaging.

## Method

### Animal model

For the time course model, we used ten commercially bought C57BL/6 female mice, eight weeks old (Charles River; Willington, USA). Each mouse received 8 contrast-enhanced micro-CT scans over a 2 week period. For the tumour model, we used twelve commercially bought C57BL/6 female mice, eight weeks old (Charles River; Willington, USA). The mice ranged in weight from 18 to 20 g. All the mice lived in a 12 h light–dark cycle in a conventional, pathogen-free animal facility with free access to food and water. All animal work was performed under ethics approval (#19-0097) obtained from the University of British Columbia Animal Care Committee. All animal work was performed in accordance with the approved protocol, and the guidelines and best practices outlined by the Canadian Council on Animal Care (CCAC) and ARRIVE.

The B16F10 murine melanoma cell line was revived from −80 °C and grown as a monolayer culture in DMEM-10% fetal bovine serum (ThermoFisher Scientific). The cells were kept in a 37 °C incubator. The twelve mice surgically received a hepatic portal vein injection of 2.5 × 10^4^ B16F10 cells in 25 µL of PBS by an animal technician and were monitored daily. To observe the range of tumor sizes and tumor burdens of B16F10 cells the tumor cells were allowed to grow for different lengths of time before the mice were imaged. One group of six mice were monitored for 3 wks and the other groups of six mice were monitored for 2 wks after their tumor injection surgery.

### In vivo micro-computed tomography imaging

Each mouse was anaesthetized in an induction box with 5% isoflurane in O_2_ then received a single tail vein injection before being transferred to the scanning table in the in vivo micro-CT scanner. Half of the mice were injected once with a tail vein dosage of 0.01 mL/g of Fenestra VC and the remaining mice were injected with 0.005 mL/g of Fenestra HDVC using a 1 mL, 29-gauge needle. Immediately after the tail vein injection, the mouse was transferred to the scanning table in the in vivo micro-CT where it was maintained under anesthesia at 2% isoflurane in O_2_ using a nosecone and given oil-based eye lube to prevent its eyes from drying (Purealube® Ophthalmic Ointment, Dechra; Texas, USA). The healthy mice in the time course study were imaged pre-contrast, post-contrast, at 8, 24, 48 and 72 h, and at 1 and 2 weeks post-injection. All the tumour-bearing mice were imaged at five time points after their tumor implanting surgery: pre-contrast, post-contrast 0, 24, 48 and 72 h.

Micro-CT images were taken using the in vivo micro-CT scanner eXplore CT 120 (TriFoil Imaging; Chatsworth, USA) at 70 kVp and 50 mA. The images were reconstructed with a filtered back-projection algorithm at 100 µm voxel spacing and filtered with an edge-preserving bilateral filter coded in Matlab to reduce noise (MATLAB 2014a, The MathWorks, Natick; Massachusetts, USA).

### In vivo image analysis

The filtered and reconstructed in vivo micro-CT images were analyzed for the greyscale values of the seven organs of interest at the different time points. The seven organs measured were left leg muscle, liver, bladder, kidney, spleen, right atrium and vena cava, along with air outside the animal to ensure correct calibration in HU. The mean greyscale values and standard deviations in HU were measured in MicroView (version ABA2.2, TriFoil Imaging; Chatsworth, USA) using 1 mm^3^ ROI for all the organs except the vena cava. The vena cava was measured with an ROI of 0.3 mm × 1 mm × 1.5 mm.

The liver tumors were monitored in the in vivo micro-CT images over 72 h post-injection. The volumes of all tumor nodules that grew within the liver with identifiable borders were measured. Among the tumor nodules and tumor masses measured, the tumor that could be confidently measured for its volume was recorded.

### Liver excision

The tumour-bearing mice were euthanized following the post-injection 72 h in vivo micro-CT scans by placing the mice in an induction box flowing with constant 5% isoflurane in O_2_ until they stopped breathing. The abdomen was opened, and the liver was removed from the mouse, then fixed in 10% formalin.

### Ex vivo micro-computed tomography imaging and analysis

The fixed livers were placed individually in 50 mL Falcon tubes for imaging using the micro-CT high resolution specimen scanner (µCT 100, SCANCO Medical; Brüttisellen, Switzerland). Images were obtained at 90 kVp, 200 µA and 17.2 µm voxel spacing, with scan times ranging between 45 and 98 min depending on liver size.

The total tumor burden for each mouse was measured in MicroView. The volume of the entire liver including vasculature and tumor against the black background was measured using a seeded region growing algorithm with a threshold value of 1640 for all the mice. Then the liver parenchyma was measured in each excised liver with a higher threshold value to identify the contrast-enhanced tissue, ranging between 2171 and 2350 to account for varying amounts of tumour in each mouse. The difference between these volumes (whole organ—enhanced parenchyma) represents the tumours and the vasculature. To estimate the vasculature, we identified one mouse that had a single tumour fully enclosed within the parenchyma that could be manually segmented. Therefore the volume of the vasculature for this mouse was calculated using Eq. 1, where VV is volume of the vasculature, VL is volume of the entire liver organ, VP is the volume of the contrast enhanced parenchyma, and VT is the tumour volume.

1 $$VV=VL-VP-VT$$

Assuming the vasculature size does not change across mice of similar weight and age the volume of the vasculature was used to estimate the tumour volume for the remaining mice, where multiple tumours made it difficult to clearly identify the tumours and the vasculature.

Using the estimated total tumor volume, the percentage of total tumor burden in the liver of each mouse can be calculated from the ex vivo images (Eq. 2).

2 $$Total tumor burden \%=\frac{VT }{VL } \times 100$$

### Statistical analysis

The mean greyscale values and standard deviations for the five mice given the same CA were averaged. The average values for the five mice injected with 0.005 mL/g of Fenestra HDVC were compared with the average values for the five mice given 0.01 mL/g of Fenestra VC for statistical analysis. Statistical analysis was performed using a two-way ANOVA and Sidak test with a p-value of 0.05 in PRISM (Prism v. 9.3.1, GraphPad Software, Inc., San Diego, CA). A power calculation was done retrospectively, and 5 mice per contrast agent provided a sufficient sample size for > 90% confidence with α = 0.05.

## Data Availability

The raw datasets used in this study are very large 3D images and therefore is impractical to submit as supplemental material. The datasets used and/or analysed during the current study are available from the corresponding author on reasonable request.
